# Coercion and force in psychiatry: when young doctors become forced to use force

**DOI:** 10.1192/j.eurpsy.2026.10280

**Published:** 2026-07-31

**Authors:** J. M. Zadicoff De Martin

**Affiliations:** Akut søjlen, Psykiatrisk Center Glostrup, Copenhagen, Denmark

## Abstract

**Abstract:**

As a young doctor, it is challenging to decide when coercion or forceful measures are necessary, as well as when the use of force follows the principle of “less harm”. This is because there are a wide spectrum of opinions, criteria,ethical questions, moral values, and laws which one must consider, and which can be difficult to understand and balance without a great amount of prior experience.

Every medicine has primary effects and side effects, and every intervention has potentially positive benefits and negative implications. As psychiatric doctors we have the obligation to not do harm, a moral duty to help others, and a lawful compulsion to use the minimum harmful measure when needed. The decision to use coercion is complicated to navigate as a young doctor in training with potentially limited experience in making such morally and legally complex decisions. We are forced to make fast decisions when an acute situation arises, where we must grapple with many considerations, including: external input and pressure, internal ethical questions about what is right and wrong, utilitarian questions about what is the best and least harmful measure to take to help our patients, unintended consequences of our decisions for the patients recovery and wellbeing and the safety of ourselves and colleagues, and the possible lawful implications of the actions and measures taken.

Furthermore, young doctors face pressure from colleagues and care personnel. Thus, when making treatment decisions, one must consider not only their own opinion, but that of potentially more senior colleagues whose ideas, methods, and approaches to the concept of “less harm” may be different. The path of educating ourselves and finding our own criteria and methods of work is long and complicated, which can lead young doctors to feel as though they are ”walking in a jungle” of uncertainty, confusion, frustration, fear and contradiction. This especially challenging when deciding on cases involving coercion and force.

This talk is based largely on personal experiences as well as screening among young doctors both within and outside of psychiatry using both personal and group interviews and surveys. Results will be contextualized with existing academic studies on the topic of coercion in psychiatry.

The aim of this talk is to present the different challenges a young doctor faces while working in a psychiatric institution, opening a debate about the concept of “less harm” in the context of an acute situation, exploring the uncertainties and ethical values that a young doctor faces on a daily basis whilst performing their job and discuss different proposals about how to reduce the amount of coercive messures.

**Disclosure of Interest:**

None Declared

